# Association between physical activity levels, sedentary time, and mild cognitive impairment in older adults

**DOI:** 10.3389/fpubh.2026.1723009

**Published:** 2026-02-10

**Authors:** Wei Chen, Lei Zhang, Palida Abulizi, Ting Zou, Xuan Xiang, Ruikai Wu, Xiaohui Zhou

**Affiliations:** Department of Geriatrics, First Affiliated Hospital of Xinjiang Medical University, Urumqi, Xinjiang, China

**Keywords:** dose–response relationship, mild cognitive impairment, older adults, physical activity, sedentary time

## Abstract

**Background:**

Research indicates that mild cognitive impairment (MCI) in older adults is associated with physical activity levels (PAL) and sedentary behavior duration. However, the precise nature of the relationships between these factors and MCI warrants further investigation.

**Methods:**

A cross-sectional survey was conducted from August to October 2025 using cluster sampling in community settings, involving 1,465 older adults. Cognitive function was assessed using the Montreal Cognitive Assessment (MoCA). PAL were assessed using the International Physical Activity Questionnaire-Short Form (IPAQ-SF), while sedentary time was self-reported. Logistic regression models were employed to analyze the associations between PAL, sedentary time, and MCI. Restricted cubic spline (RCS) analysis was used to further explore the dose–response relationships. Sensitivity analyses were also performed to validate the observed associations.

**Results:**

Logistic regression analysis revealed that the second and fourth quartiles of PAL (vs. the first quartile) were associated with a significantly reduced risk of MCI (*OR* = 0.544, *p* < 0.05 and *OR* = 0.345, *p* < 0.05, respectively). The second quartile of sedentary time (vs. the first quartile) was also associated with a significantly lower MCI risk (*OR* = 0.561, *p* < 0.05). RCS analysis showed that as PAL increased, the risk of MCI gradually decreased, with the most pronounced cognitive benefit observed at approximately 1,485 MET-min/wk. However, when PAL exceeded 4,000 MET-min/wk., the MCI risk tended to increase. For sedentary time, MCI risk initially decreased and then increased with longer duration. The lowest risk was observed at around 150 min/day, with risk beginning to rise after exceeding 200 min/day. Sensitivity analysis confirmed that the relationships between PAL, sedentary time, and MCI remained robust.

**Conclusion:**

Both physical activity and sedentary time are closely associated with the incidence of MCI in older adults. Maintaining a weekly PAL between 1,485 and 4,000 MET-min/wk. and limiting daily sedentary time to under 200 min may help reduce the risk of MCI.

## 1 Introduction

Population aging represents a significant demographic trend in the 21st century, impacting various domains including economics, society, culture, and healthcare. According to the United Nations’ World Population Prospects 2024 (1), the average age of the world’s population is also increasing. By the end of the 2070s, the number of people aged 65 and above is expected to exceed the number of people under the age of 18. World Health Organization (WHO) data indicate that the global population aged 60 and above is expected to grow from 1 billion in 2019 to 2.1 billion by 2050, accounting for 22% of the world’s total population. The WHO’s Global Report on Ageing and Health (2) highlights population aging as a major global challenge. Aging not only alters the disease spectrum, making chronic non-communicable diseases a primary disease burden, but also intensifies issues such as geriatric syndromes, disability, and cognitive impairment, posing comprehensive and multi-level challenges to healthcare systems.

Alzheimer’s disease (AD) and related dementias impose a heavy burden on society, families, and healthcare systems (3, 4). The Global Impact of Dementia 2013–2050 report notes (5) approximately 7.7 million new dementia cases annually, yet there remains no definitive disease-modifying treatment. The Lancet Commission’s 2024 report on dementia prevention, intervention, and care (6) states that controlling 14 modifiable risk factors, including physical activity, could prevent or delay up to 40% of dementia cases globally. Research in The Lancet found (7) that 3% of dementia cases could be prevented by increasing free-living physical activity levels. Mild cognitive impairment (MCI) represents a transitional state between normal aging and dementia, serving as a critical ‘intervention window’ for dementia prevention. Individuals with MCI maintain normal basic daily activities but may experience slight impairments in complex instrumental activities, not meeting the diagnostic criteria for dementia. As a significant prodromal stage, individuals with MCI have a higher risk of progressing to dementia, with an annual conversion rate between 10 and 15% (8, 9), yet this period also represents a key “window of opportunity.”

Given the heterogeneity of MCI etiology and the multiplicity of risk factors, non-pharmacological interventions for MCI have become a major focus in international research. The most advanced concept in expert consensus involves implementing multi-domain integrated interventions, combining exercise training, cognitive training, dietary intervention, lifestyle and risk factor management, psychosocial intervention, and social participation, which can effectively delay progression from MCI to dementia. Among these, exercise intervention is considered the most economical and convenient approach. The WHO’s 2020 Guidelines on Physical Activity and Sedentary Behavior (10) recommend that older adults (≥65 years) engage in multi-component physical activity, focusing on functional balance and strength training, at least three times per week as part of weekly moderate-intensity aerobic activity, to help prevent cognitive decline. The guidelines also emphasize the importance of limiting sedentary time and replacing it with physical activity of any intensity. As age increases, physical activity levels (PAL) in older adults tend to decline (11). Older adults often prefer staying at home or walking only near their residences, which may lead to insufficient physical activity. A study by Chan et al. (12) found an overall prevalence of physical inactivity of 48.8% among older adults aged 60 and above. The WHO’s Guidelines on Risk Reduction of Cognitive Decline and Dementia (13) and the Lancet Commission’s Dementia prevention, intervention, and care: 2020 report (14) identify regular physical activity as an effective strategy for reducing the risk of cognitive decline and AD. They recommend interrupting prolonged sitting as an essential component of primary prevention and MCI management, suggesting 2–3 min of activity after every 30 min of sitting. WHO recommends physical exercise for older adults with normal cognition and those with MCI to reduce the risk of cognitive decline. It advises that adults aged 65 and above should engage in at least 150 min of moderate-intensity aerobic physical activity, or at least 75 min of vigorous-intensity aerobic activity, or an equivalent combination per week, to achieve the recommended level (600 metabolic equivalent minutes per week) (15–17).

Older adults are the age group with the lowest levels of physical activity and spend a significant portion of their day being sedentary (18). Sedentary behavior is defined as any waking behavior characterized by an energy expenditure ≤1.5 METs (Metabolic equivalent, MET) while in a sitting, reclining, or lying posture (19, 20). Research has found that longer sedentary time among older adults is associated with a higher incidence of all-cause dementia (21). Our previous study based on the China Health and Retirement Longitudinal Study found (22) that light to moderate physical activity reduces the risk of MCI in older adults. Xinjiang region, located in the heart of the Eurasian continent (≥2,500 km from the nearest ocean) and covering one-sixth of China’s land area, is the region farthest from the ocean in the world. This study aims to investigate the relationships between PAL, sedentary time, and MCI among community-dwelling older adults in Xinjiang, Northwest China, and to define recommended ranges for PAL and sedentary time to inform MCI prevention and management strategies.

## 2 Materials and methods

### 2.1 Data source

This cross-sectional study was conducted from August to October 2025 using a cluster sampling method. A total of 1,465 adults aged 60 and above were recruited from three communities in Jimsar County, Xinjiang. Inclusion criteria were: (1) age ≥60 years; (2) residence in the survey area for ≥1 year; (3) ability to cooperate in completing questionnaires. The study was approved by the Ethics Committee of the First Affiliated Hospital of Xinjiang Medical University (Approval No.: 240528–01), complied with the principles of the Declaration of Helsinki, and all participants provided informed consent.

### 2.2 Main variables

#### 2.2.1 Mild cognitive impairment

Cognitive function was assessed using the Montreal Cognitive Assessment (MoCA). The scale covers executive function, verbal fluency, orientation, calculation, abstraction, delayed recall, visuospatial ability, naming, attention, and concentration, with a total score ranging from 0 to 30. Lower scores indicate poorer cognitive function. MCI screening cutoffs were adjusted for education level: ≤13 for illiteracy, ≤19 for primary school, and ≤24 for junior high school and above (23, 24). Based on these criteria, 1,308 participants were classified as cognitively normal and 157 as having MCI. For sensitivity analysis, an adjusted MoCA threshold (adding 1 point for ≤12 years of education) was also applied, resulting in 1352 participants classified as normal and 113 as having MCI.

#### 2.2.2 Physical activity level assessment

PAL were assessed using the International Physical Activity Questionnaire-Short Form (IPAQ-SF) (25), a widely used tool with demonstrated reliability and validity. MET values were assigned to each activity: 3.3 for walking, 4.0 for moderate-intensity activities, and 8.0 for vigorous-intensity activities. Weekly total energy expenditure was calculated as PAL (MET-min/wk) using the formula: MET value × daily activity duration (minutes) × weekly activity days (17, 26). PAL was categorized into quartiles for analysis: Q1 (<1039.5 MET-min/wk), Q2 (1039.5 ≤ PAL < 1,485 MET-min/wk), Q3 (1,485 ≤ PAL < 2079 MET-min/wk), and Q4 (≥2079 MET-min/wk).

#### 2.2.3 Sedentary time

Sedentary time was self-reported, referring to time spent sitting during waking hours at home, while traveling, or with friends, including activities such as sitting at a desk, traveling in vehicles, reading, playing cards, watching TV, or using a computer, excluding sleep time. Sedentary time was categorized into quartiles: Q1 (<120 min/day), Q2 (120 ≤ time < 150 min/day), Q3 (150 ≤ time < 200 min/day), and Q4 (≥200 min/day).

#### 2.2.4 Activities of daily living

The Activities of Daily Living (ADL) scale was used to assess functional disability. ADL was divided into Basic ADL (BADL: toileting, eating, dressing, controlling excretion, getting in/out of bed, bathing) and Instrumental ADL (IADL: shopping, using the telephone, cooking, housekeeping, managing medications, handling finances). Participants were asked about difficulties performing these tasks. An ADL disability score (range 0–11) was calculated by summing responses across the 12 items. Participants reporting difficulty in any item were classified as having ADL impairment.

#### 2.2.5 Depression

Depression was assessed using the 9-item Patient Health Questionnaire (PHQ-9), a valid and reliable self-report scale based on DSM-IV criteria. Participants rated the frequency of symptoms over the past 2 weeks (0=“not at all” to 3=“nearly every day”). A total score ≥10 indicated probable depression.

#### 2.2.6 Covariates

Based on the study design, variables of different dimensions were assessed. For binary categorical variables, values of 0 and 1 were assigned. For categorical variables with three or more levels, incremental values (e.g., 0, 1, 2) were assigned. The specific variables included were:

Demographics: Age, sex, education level, marital status, pre-retirement occupation, living arrangement, and days living alone.
Lifestyle habits: Smoking, alcohol consumption, dietary habits, sleep quality, and central obesity.
Medical history: Family history of dementia, history of head injury.
Chronic diseases: Hypertension, dyslipidemia, diabetes or elevated blood glucose, chronic lung disease, heart disease (e.g., myocardial infarction, coronary heart disease, angina, congestive heart failure), stroke, gastric disease, arthritis or rheumatism.

### 2.3 Statistical analysis

Statistical analyses were performed using R software version 4.3.0. Continuous data with non-normal distributions are presented as median (interquartile range) [M (Q₁, Q₃)], and group comparisons were made using non-parametric tests. Categorical data are presented as *n* (%), and group comparisons were made using the chi-square test or Fisher’s exact test. Binary logistic regression was used to analyze the relationships between PAL, sedentary time, and MCI. Six models were constructed:

Model 1: Unadjusted.
Model 2: Adjusted for demographics and lifestyle factors significantly different in univariate analysis.
Model 3: Adjusted for demographics, lifestyle factors, and chronic diseases significantly different in univariate analysis.
Model 4: Based on the adjusted MoCA threshold MCI grouping, unadjusted.
Model 5: Based on the adjusted MoCA threshold MCI grouping, adjusted as per Model 2.
Model 6: Based on the adjusted MoCA threshold MCI grouping, adjusted as per Model 3.

Restricted cubic spline (RCS) analysis using the “ggrcs” package was performed to evaluate the dose–response relationships between PAL, sedentary time, and MCI, with 4 nodes and the median of the exposure variable set as the reference point. Results are presented as odds ratios (OR) with 95% confidence intervals (CI). A two-sided *p* value < 0.05 was considered statistically significant.

## 3 Results

### 3.1 Participant characteristics

The study included 1,465 participants. According to the standard MoCA criteria, 1,308 (89.28%) were cognitively normal, and 157 (10.72%) had MCI. Significant differences (*p* < 0.05) were observed between the normal and MCI groups in PAL, sedentary time, age, education level, days living alone, central obesity, dietary habits, sleep quality, ADL, depression, dyslipidemia, stroke, gastric disease, and arthritis/rheumatism. No significant differences (*p* > 0.05) were found for sex, marital status, pre-retirement occupation, family history of dementia, history of head injury, living arrangement, smoking, alcohol consumption, hypertension, diabetes, chronic lung disease, or heart disease (Table 1).

**Table 1:** Comparison of general characteristics of participants.

Variables	Total (*n* = 1,465)	Cognitive normal group (*n* = 1,308)	MCI group (*n* = 157)	Statistic	*p*
Physical activity, *n* (%)				*χ*^2^ = 46.14	**<0.001**
Q1	291 (19.86)	229 (17.51)	62 (39.49)		
Q2	431 (29.42)	388 (29.66)	43 (27.39)		
Q3	202 (13.79)	184 (14.07)	18 (11.46)		
Q4	541 (36.93)	507 (38.76)	34 (21.66)		
Sedentary, *n* (%)				*χ*^2^ = 21.82	**<0.001**
Q1	290 (19.80)	251 (19.19)	39 (24.84)		
Q2	417 (28.46)	388 (29.66)	29 (18.47)		
Q3	323 (22.05)	300 (22.94)	23 (14.65)		
Q4	435 (29.69)	369 (28.21)	66 (42.04)		
Age, *M* (*Q_1_*, *Q_3_*)	71.00 (66.00,76.00)	71.00 (66.00,76.00)	72.00 (67.00,78.00)	*Z* = −2.23	**0.026**
Gender, *n* (%)				*χ*^2^ = 0.87	0.352
Male	658 (44.91)	582 (44.50)	76 (48.41)		
Woman	807 (55.09)	726 (55.50)	81 (51.59)		
Educational level, *n* (%)				*χ*^2^ = 7.75	**0.021**
Illiterate	363 (24.78)	315 (24.08)	48 (30.57)		
Primary school	506 (34.54)	445 (34.02)	61 (38.85)		
Junior high school and above	596 (40.68)	548 (41.90)	48 (30.57)		
Marital status, *n* (%)				–	0.671
Married, living with spouse	1,197 (81.71)	1,073 (82.03)	124 (78.98)		
Married, but temporarily not living with spouse due to work or other reasons	59 (4.03)	52 (3.98)	7 (4.46)		
Separation	18 (1.23)	15 (1.15)	3 (1.91)		
Divorced	25 (1.71)	21 (1.61)	4 (2.55)		
Widowed	165 (11.26)	146 (11.16)	19 (12.10)		
Never married	1 (0.07)	1 (0.08)	0 (0.00)		
Pre-retirement occupation, *n* (%)				*χ*^2^ = 3.09	0.686
Civil servants or enterprises, and institutions	130 (8.87)	117 (8.94)	13 (8.28)		
Professional technical personnel	124 (8.46)	113 (8.64)	11 (7.01)		
Office personnel or staff	164 (11.19)	144 (11.01)	20 (12.74)		
Commercial services	19 (1.30)	15 (1.15)	4 (2.55)		
Agricultural, forestry, animal husbandry, and fishery production personnel	878 (59.93)	784 (59.94)	94 (59.87)		
Production and transportation equipment operators	150 (10.24)	135 (10.32)	15 (9.55)		
Residential style, *n* (%)				*χ*^2^ = 1.59	0.452
Spouse cohabitation	1,276 (87.10)	1,143 (87.39)	133 (84.71)		
Shared residence for children	116 (7.92)	103 (7.87)	13 (8.28)		
Living alone	73 (4.98)	62 (4.74)	11 (7.01)		
Number of days living alone, *n* (%)				*χ*^2^ = 10.96	**0.027**
0	676 (46.14)	607 (46.41)	69 (43.95)		
1 ~ 29	622 (42.46)	560 (42.81)	62 (39.49)		
30 ~ 59	53 (3.62)	42 (3.21)	11 (7.01)		
60 ~ 90	19 (1.30)	19 (1.45)	0 (0.00)		
90 and above	95 (6.48)	80 (6.12)	15 (9.55)		
Central obesity, *n* (%)				*χ*^2^ = 4.99	**0.026**
No	244 (16.66)	208 (15.90)	36 (22.93)		
Yes	1,221 (83.34)	1,100 (84.10)	121 (77.07)		
Smoking habits, *n* (%)				*χ*^2^ = 0.02	0.889
No	1,265 (86.35)	1,130 (86.39)	135 (85.99)		
Yes	200 (13.65)	178 (13.61)	22 (14.01)		
Drinking habits, *n* (%)				*χ*^2^ = 0.29	0.592
No	1,248 (85.19)	1,112 (85.02)	136 (86.62)		
Yes	217 (14.81)	196 (14.98)	21 (13.38)		
Eating habits, *n* (%)				*χ*^2^ = 9.74	**0.008**
Balanced meat and vegetables	1,365 (93.17)	1,228 (93.88)	137 (87.26)		
Mainly meat dishes	62 (4.23)	50 (3.82)	12 (7.64)		
Mainly vegetarian	38 (2.59)	30 (2.29)	8 (5.10)		
Sleep quality, *n* (%)				*χ*^2^ = 20.18	**<0.001**
Very good	491 (33.52)	436 (33.33)	55 (35.03)		
Better	506 (34.54)	468 (35.78)	38 (24.20)		
Poor	359 (24.51)	319 (24.39)	40 (25.48)		
Very poor	109 (7.44)	85 (6.50)	24 (15.29)		
ADL, *n* (%)				*χ*^2^ = 53.11	**<0.001**
Normal	1,096 (74.81)	1,016 (77.68)	80 (50.96)		
Obstacle	369 (25.19)	292 (22.32)	77 (49.04)		
Family history of dementia, *n* (%)				*χ*^2^ = 0.99	0.319
No	1,452 (99.11)	1,298 (99.24)	154 (98.09)		
Yes	13 (0.89)	10 (0.76)	3 (1.91)		
Head trauma, *n* (%)				*χ*^2^ = 0.00	1
No	1,448 (98.84)	1,293 (98.85)	155 (98.73)		
Yes	17 (1.16)	15 (1.15)	2 (1.27)		
Depression, *n* (%)				*χ*^2^ = 29.70	**<0.001**
No	950 (64.85)	879 (67.20)	71 (45.22)		
Yes	515 (35.15)	429 (32.80)	86 (54.78)		
Hypertension, *n* (%)				*χ*^2^ = 0.11	0.735
No	514 (35.09)	457 (34.94)	57 (36.31)		
Yes	951 (64.91)	851 (65.06)	100 (63.69)		
Abnormal blood lipids, *n* (%)				*χ*^2^ = 16.64	**<0.001**
No	1,446 (98.70)	1,297 (99.16)	149 (94.90)		
Yes	19 (1.30)	11 (0.84)	8 (5.10)		
Diabetes, *n* (%)				*χ*^2^ = 0.16	0.693
No	852 (58.16)	763 (58.33)	89 (56.69)		
Yes	613 (41.84)	545 (41.67)	68 (43.31)		
Chronic lung diseases, *n* (%)				-	0.549
No	1,458 (99.52)	1,302 (99.54)	156 (99.36)		
Yes	7 (0.48)	6 (0.46)	1 (0.64)		
Heart disease, *n* (%)				*χ*^2^ = 1.81	0.178
No	1,038 (70.85)	934 (71.41)	104 (66.24)		
Yes	427 (29.15)	374 (28.59)	53 (33.76)		
Stroke, *n* (%)				*χ*^2^ = 65.94	**<0.001**
No	1,436 (98.02)	1,296 (99.08)	140 (89.17)		
Yes	29 (1.98)	12 (0.92)	17 (10.83)		
Stomach diseases, *n* (%)				*χ*^2^ = 7.52	**0.006**
No	1,442 (98.43)	1,292 (98.78)	150 (95.54)		
Yes	23 (1.57)	16 (1.22)	7 (4.46)		
Arthritis or rheumatism, *n* (%)				–	**0.019**
No	1,459 (99.59)	1,305 (99.77)	154 (98.09)		
Yes	6 (0.41)	3 (0.23)	3 (1.91)		

Bold values indicate *P* < 0.05; Fisher’s exact test.

### 3.2 Logistic regression analysis of the association between PAL, sedentary time, and MCI in older adults

MCI was the dependent variable, and PAL and sedentary time were the independent variables. Variables with significant univariate differences (*p* < 0.05) were included in the regression models to assess their relationships (variable assignments shown in Table 2).

**Table 2:** Variable coding table.

Variables	Assignment table
Physical activity	1 = Q1, 2 = Q2, 3 = Q3, 4 = Q4
Sedentary	1 = Q1, 2 = Q2, 3 = Q3, 4 = Q4
Age	Continuous Variable
Education level	1 = Illiterate, 2 = Primary School, 3 = Junior High School And Above
Number of days living alone	1 = 0, 2 = 1 ~ 29, 3 = 30 ~ 59, 4 = 60 ~ 89, 5 = 90 And Above
Central obesity	1 = No, 2 = Yes
Dietary habits	1 = Balanced Meat And Vegetable Diet, 2 = Mainly Meat Based Diet, 3 = Mainly Vegetarian Diet
Sleep quality	1 = Very Good, 2 = Good, 3 = Poor, 4 = Very Poor
ADL	1 = Normal, 2 = Obstacle
Depression	1 = No, 2 = Yes
Dyslipidemia	1 = No, 2 = Yes
Stroke	1 = No, 2 = Yes
Gastric diseases	1 = No, 2 = Yes
Arthritis or rheumatism	1 = No, 2 = Yes

The variance inflation factor (VIF) for all covariates was <5, and tolerance was far >0.1, indicating no significant multicollinearity (Table 3).

**Table 3:** Multicollinearity test.

Variable	Model 1 collinearity statistics		Variable	Model 2 collinearity statistics		Variable	Model 3 collinearity statistics		Variable	Model 4 collinearity statistics		Variable	Model 5 collinearity statistics		Variable	Model 6 collinearity statistics	
	Tolerance	VIF		Tolerance	VIF		Tolerance	VIF		Tolerance	VIF		Tolerance	VIF		Tolerance	VIF
PAL	/	/	PAL	0.838	1.193	PAL	0.813	1.230	PAL	/	/	PAL	0.838	1.193	PAL	0.813	1.230
			Age	0.547	1.828	Age	0.535	1.868				Age	0.547	1.828	Age	0.535	1.868
			Education level	0.564	1.773	Education level	0.554	1.807				Education level	0.564	1.773	Education level	0.554	1.807
			Number of days living alone	0.932	1.073	Number of days living alone	0.918	1.089				Number of days living alone	0.932	1.073	Number of days living alone	0.918	1.089
			Central obesity	0.988	1.012	Central obesity	0.963	1.039				Central obesity	0.988	1.012	Central obesity	0.963	1.039
			Dietary habits	0.922	1.085	Dietary habits	0.88	1.136				Dietary habits	0.922	1.085	Dietary habits	0.88	1.136
			Sleep quality	0.933	1.072	Sleep quality	0.911	1.098				Sleep quality	0.933	1.072	Sleep quality	0.911	1.098
			ADL	0.792	1.262	ADL	0.747	1.339				ADL	0.792	1.262	ADL	0.747	1.339
						Depression	0.813	1.230							Depression	0.813	1.230
						Dyslipidemia	0.951	1.052							Dyslipidemia	0.951	1.052
						Stroke	0.926	1.080							Stroke	0.926	1.080
						Gastric diseases	0.963	1.038							Gastric diseases	0.963	1.038
						Arthritis or rheumatism	0.984	1.016							Arthritis or rheumatism	0.984	1.016
Sedentary	/	/	Sedentary	0.884	1.132	Sedentary	0.842	1.188	Sedentary	/	/	Sedentary	0.884	1.132	Sedentary	0.842	1.188
			Age	0.547	1.829	Age	0.536	1.864				Age	0.547	1.829	Age	0.536	1.864
			Education level	0.553	1.808	Education level	0.545	1.833				Education level	0.553	1.808	Education level	0.545	1.833
			Number of days living alone	0.922	1.084	Number of days living alone	0.911	1.098				Number of days living alone	0.922	1.084	Number of days living alone	0.911	1.098
			Central obesity	0.969	1.032	Central obesity	0.949	1.053				Central obesity	0.969	1.032	Central obesity	0.949	1.053
			Dietary habits	0.934	1.071	Dietary habits	0.888	1.126				Dietary habits	0.934	1.071	Dietary habits	0.888	1.126
			Sleep quality	0.934	1.071	Sleep quality	0.908	1.101				Sleep quality	0.934	1.071	Sleep quality	0.908	1.101
			ADL	0.805	1.242	ADL	0.756	1.323				ADL	0.805	1.242	ADL	0.756	1.323
						Depression	0.797	1.254							Depression	0.797	1.254
						Dyslipidemia	0.95	1.053							Dyslipidemia	0.95	1.053
						Stroke	0.928	1.078							Stroke	0.928	1.078
						Gastric diseases	0.965	1.036							Gastric diseases	0.965	1.036
						Arthritis or rheumatism	0.982	1.018							Arthritis or rheumatism	0.982	1.018

Model 1: Unadjusted. Model 2: Model 1 + Age, Education Level, Days Living Alone, Central Obesity, Dietary Habits, Sleep Quality, ADL. Model 3: Model 1 + Age, Education Level, Days Living Alone, Central Obesity, Dietary Habits, Sleep Quality, ADL, Depression, Dyslipidemia, Stroke, Gastric Disease, Arthritis/Rheumatism. Model 4: Adjusted Moca Threshold-Based MCI Group, Unadjusted. Model 5: Model 4 + Age, Education Level, Days Living Alone, Central Obesity, Dietary Habits, Sleep Quality, ADL. Model 6: Model 4 + Age, Education Level, Days Living Alone, Central Obesity, Dietary Habits, Sleep Quality, ADL, Depression, Dyslipidemia, Stroke, Gastric Disease, Arthritis/Rheumatism. VIF, Variance Inflation Factor.

In the unadjusted logistic regression model (Model 1), the second [*OR* = 0.409 (*95%CI*: 0.268–0.624)], third [*OR* = 0.361 (*95%CI*: 0.206–0.632)], and fourth [*OR* = 0.248 (*95%CI*: 0.159–0.387)] PAL quartiles were associated with significantly reduced MCI risk compared to the first quartile. Similarly, the second [*OR* = 0.481 (*95%CI*: 0.290–0.798)] and third [*OR* = 0.493 (*95%CI*: 0.287–0.848)] sedentary time quartiles were associated with lower risk compared to the first quartile.

After adjusting for demographic and lifestyle factors (Model 2), the second [*OR* = 0.497 (*95%CI*: 0.296–0.834)] and fourth [*OR* = 0.318 (*95%CI*: 0.184–0.549)] PAL quartiles, and the second sedentary time quartile [*OR* = 0.477 (*95%CI*: 0.278–0.817)], remained significantly associated with reduced MCI risk.

After further adjustment for chronic diseases (Model 3), the associations for the second [*OR* = 0.544 (*95%CI*: 0.318–0.930)] and fourth [*OR* = 0.345 (*95%CI*: 0.194–0.614)] PAL quartiles, and the second sedentary time quartile [*OR* = 0.561 (*95%CI*: 0.320–0.982)], persisted.

Sensitivity analyses using the adjusted MoCA threshold f*or* MCI classification (Models 4, 5, 6) yielded consistent results, confirming the robustness of the associations between PAL, sedentary time, and MCI (Table 4).

**Table 4:** Logistic regression analysis of the associations between pal, sedentary time, and MCI in older adults.

Variable	Subgroup	Model 1	Model 2	Model 3	Model 4	Model 5	Model 6
PAL	Q1	1.000 (Reference)	1.000 (Reference)	1.000 (Reference)	1.000 (Reference)	1.000 (Reference)	1.000 (Reference)
	Q2	0.409 (0.268–0.624)*	0.497 (0.296–0.834)*	0.544 (0.318–0.930)*	0.304 (0.183–0.505)*	0.355 (0.194–0.650)*	0.365 (0.196–0.681)*
	Q3	0.361 (0.206–0.632)*	0.647 (0.350–1.196)	0.684 (0.361–1.298)	0.340 (0.179–0.644)*	0.483 (0.239–0.976)*	0.480 (0.231–0.996)*
	Q4	0.248 (0.159–0.387)*	0.318 (0.184–0.549)*	0.345 (0.194–0.614)*	0.249 (0.151–0.411)*	0.269 (0.147–0.493)*	0.275 (0.146–0.517)*
Sedentary	Q1	1.000 (Reference)	1.000 (Reference)	1.000 (Reference)	1.000 (Reference)	1.000 (Reference)	1.000 (Reference)
	Q2	0.481 (0.290–0.798)*	0.477 (0.278–0.817)*	0.561 (0.320–0.982)*	0.280 (0.147–0.535)*	0.324 (0.164–0.639)*	0.384 (0.190–0.776)*
	Q3	0.493 (0.287–0.848)*	0.578 (0.328–1.017)	0.672 (0.370–1.218)	0.448 (0.243–0.825)*	0.548 (0.291–1.034)	0.649 (0.333–1.266)
	Q4	1.151 (0.751–1.764)	1.060 (0.648–1.735)	1.104 (0.658–1.854)	1.047 (0.654–1.677)	1.186 (0.692–2.035)	1.301 (0.736–2.300)

*p < 0.05. Model 1: Unadjusted. Model 2: Model 1 + Age, Education Level, Days Living Alone, Central Obesity, Dietary Habits, Sleep Quality, ADL. Model 3: Model 1 + Age, Education Level, Days Living Alone, Central Obesity, Dietary Habits, Sleep Quality, ADL, Depression, Dyslipidemia, Stroke, Gastric Disease, Arthritis/Rheumatism. Model 4: Adjusted Moca Threshold-Based MCI Group, Unadjusted. Model 5: Model 4 + Age, Education Level, Days Living Alone, Central Obesity, Dietary Habits, Sleep Quality, ADL. Model 6: Model 4 + Age, Education Level, Days Living Alone, Central Obesity, Dietary Habits, Sleep Quality, ADL, Depression, Dyslipidemia, Stroke, Gastric Disease, Arthritis/Rheumatism. PAL, Physical Activity Level; OR, Odds Ratio; CI, Confidence Interval.

### 3.3 Dose–response relationship between PAL and MCI

**Figure 1:**
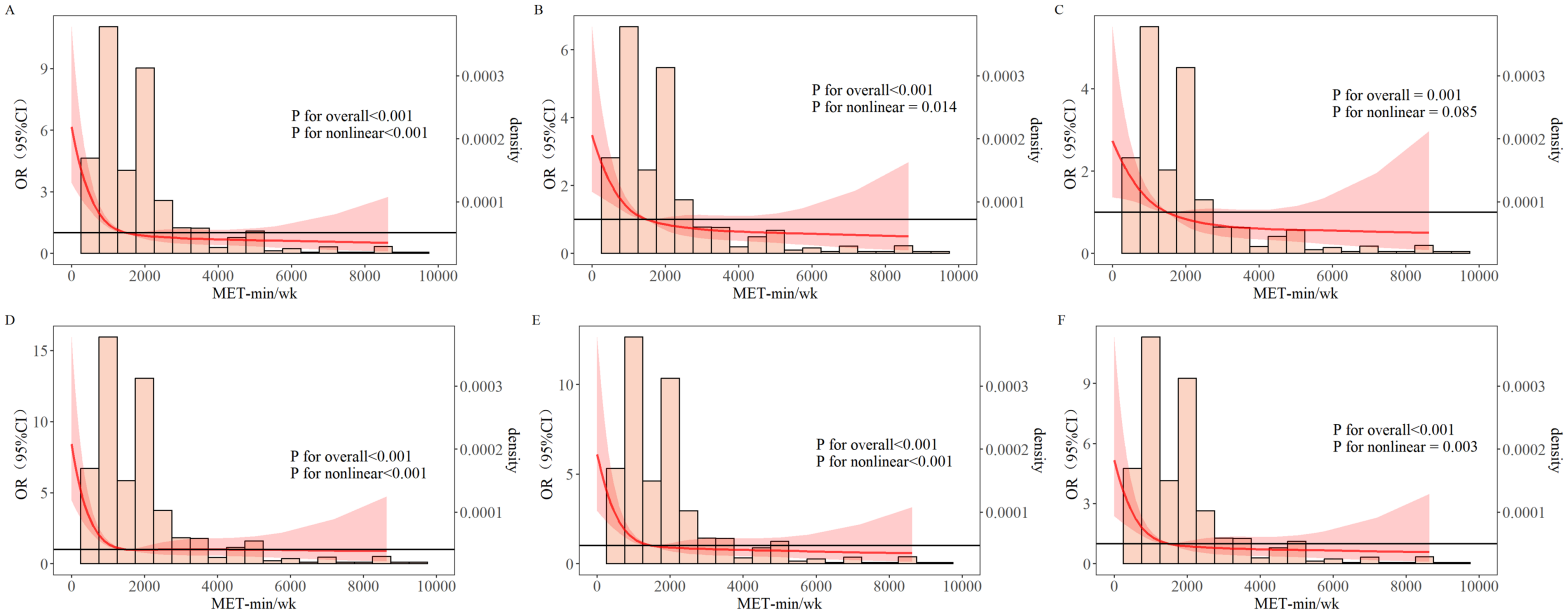
Dose–response relationship between PAL and MCI in older adults. RCS **(A)** Unadjusted. RCS **(B)** RCS A + Age, education level, days living alone, central obesity, dietary habits, sleep quality, ADL. RCS **(C)** RCS A + Age, education level, days living alone, central obesity, dietary habits, sleep quality, ADL, depression, dyslipidemia, stroke, gastric disease, arthritis/rheumatism. RCS **(D)** Adjusted Moca threshold-based MCI group, unadjusted. RCS **(E)** RCS D + Age, education level, days living alone, central obesity, dietary habits, sleep quality, ADL. RCS **(F)** RCS D + Age, education level, days living alone, central obesity, dietary habits, sleep quality, ADL, depression, dyslipidemia, stroke, gastric disease, arthritis/rheumatism. MET, Metabolic equivalent; OR, Odds ratio; CI, confidence interval.

RCS curves showed a nonlinear relationship between PAL and MCI (*P_overall_* < 0.05, *P_nonlinear_* < 0.05) (Figure 1A). The risk of MCI decreased gradually with increasing PAL, with the most benefit observed around 1,485 MET-min/wk. Beyond approximately 4,000 MET-min/wk., the risk tended to increase. After adjusting for demographic and lifestyle factors (Figure 1B), the nonlinear relationship remained (*P_overall_* < 0.05, *P_nonlinear_* < 0.05). After further adjustment for chronic diseases (Figure 1C), the relationship appeared linear (*P_overall_* < 0.05, *P_nonlinear_* > 0.05). Sensitivity analyses using the adjusted MoCA threshold (Figures 1D–F) confirmed the close association between PAL and MCI.

### 3.4 Dose–response relationship between sedentary time and MCI

**Figure 2:**
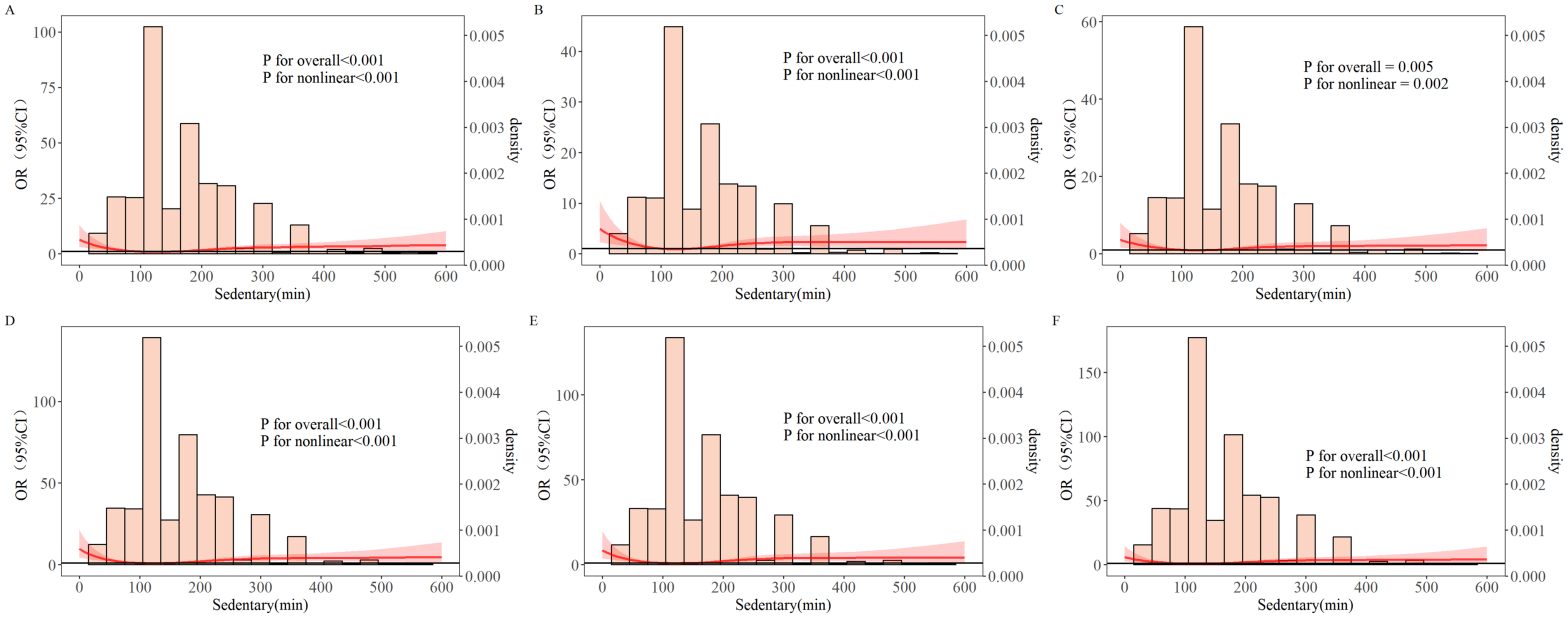
Dose–response relationship between sedentary time and MCI in older adults. RCS **(A)** unadjusted. RCS **(B)** RCS A + age, education level, days living alone, central obesity, dietary habits, sleep quality, ADL. RCS **(C)** RCS A + age, education level, days living alone, central obesity, dietary habits, sleep quality, ADL, depression, dyslipidemia, stroke, gastric disease, arthritis/rheumatism. RCS **(D)** adjusted MOCA threshold-based MCI group, unadjusted. RCS **(E)** RCS D + age, education level, days living alone, central obesity, dietary habits, sleep quality, ADL. RCS **(F)** RCS D + age, education level, days living alone, central obesity, dietary habits, sleep quality, ADL, depression, dyslipidemia, stroke, gastric disease, arthritis/rheumatism. OR, odds ratio; CI, confidence interval.

RCS curves indicated a nonlinear relationship between sedentary time and MCI (*P_overall_* < 0.05, *P_nonlinear_* < 0.05) (Figure 2A). MCI risk initially decreased with increasing sedentary time, reaching its lowest point around 150 min/day, and then increased after exceeding approximately 200 min/day. This nonlinear pattern persisted after adjusting for demographics and lifestyle factors (Figure 2B) (*P_overall_* < 0.05, *P_nonlinear_* < 0.05) and after further adjusting for chronic diseases (Figure 2C) (*P_overall_* < 0.05, *P_nonlinear_* < 0.05). Sensitivity analyses using the adjusted MoCA threshold (Figures 2D–F) corroborated the strong association.

## 4 Discussion

With the increasing global aging population and extended life expectancy, cognitive impairment imposes a significant disease and economic burden on older adults. Dementia care is costly (27, 28); US statistics show dementia patients incur over $387,000 per person in the last five years of life (29). The number of dementia cases is projected to double in coming decades, escalating associated costs. Guidelines such as those on physical activity and exercise for preventing and managing MCI and dementia (30–34) identify exercise as an effective intervention. Regular PAL is associated with a reduced conversion rate from MCI to dementia; one study reported a 15% reduction with regular physical activity (35, 36). Research reviews (37) affirm the cognitive benefits of physical activity across the lifespan and for populations with cognitive deficits. The Finnish Geriatric Intervention Study to Prevent Cognitive Impairment and Disability (FINGER) (38) demonstrated the effectiveness of multi-domain lifestyle interventions (combining diet, exercise, cognitive training, and vascular risk monitoring) on cognitive function in older adults.

In our study population, most participants had an education level of junior high school or below. However, the group exhibited habits of morning and evening walks, exercise, and square dancing. Occupations primarily involved farming and herding labor, which likely influenced MCI prevalence. Our analysis found that compared to those in the lowest PAL quartile, older adults with higher PAL had a reduced risk of MCI (OR < 1, *p* < 0.05), consistent with findings from Song et al. and others (39–50). A 5.6-year follow-up study by Verghese et al. confirmed (51) that leisure activities reduce the risk of amnestic MCI. The Australian Physical Activity and Sedentary Behavior Guidelines recommend (52) that adults aged ≥65 years engage in at least 30 min of moderate-intensity physical activity on most days, in addition to daily activities. Numerous prospective cohort studies consistently show that higher overall physical activity levels are significantly associated with a lower risk of MCI and dementia. A meta-analysis indicated that the most active older adults have approximately 30–40% lower risk of cognitive impairment compared to the least active. This suggests that older adults should enhance their PAL and improve sleep quality to bolster cognitive function.

Not only aerobic exercise (e.g., brisk walking, swimming) but also resistance training has proven benefits for specific cognitive domains like executive function (53, 54). RCS analysis revealed a nonlinear dose–response relationship between PAL and MCI. MCI risk decreased with increasing PAL, with optimal benefit around 1,485 MET-min/wk., aligning with findings by Daniel et al. (55, 56) that exercise improves cognitive function in older adults. Physiologically, appropriate physical activity duration promotes cerebral blood circulation and redistribution, enhances antioxidant effects by increasing enzyme and anti-inflammatory cytokine activity (57, 58), and elevates brain-derived neurotrophic factor (BDNF) levels (59). BDNF stimulates neuronal growth and maintenance, promotes hippocampal neurogenesis and synaptic plasticity—the cellular basis of learning and memory (60, 61). Physical activity also protects brain function by enhancing the body’s antioxidant defense system, preserving neuronal structural integrity and brain volume (brain reserve), thereby improving cognitive abilities in older adults (62–65). Regular exercise modulates systemic and central nervous system inflammation, reduces pro-inflammatory cytokines, and boosts antioxidant defenses. Animal studies suggest exercise may enhance glymphatic system function, accelerating the clearance of toxic substances like *β*-amyloid.

This study also found that the second quartile of sedentary time was associated with a lower MCI risk compared to the first quartile (OR < 1, *p* < 0.05). Cross-sectional and prospective studies indicate better neurocognitive function in more physically active individuals compared to sedentary ones (51, 66–68). RCS analysis showed a nonlinear relationship between sedentary time and MCI. Risk decreased initially with increased sedentary time but started to rise after exceeding 200 min/day, consistent with studies by Shuai Z et al. (69, 70) which found lower MCI likelihood with 1–2 h of sitting. A multinational study on MCI and sedentary behavior found each additional hour of sedentary time increased the odds of MCI by 8% (71). An evidence-based review (72) noted that long-term sedentary older adults are more prone to MCI than those engaged in physical activity or with short sedentary bouts, a trend more pronounced in Asian populations. Xie et al. (73) found a significant association between excessive sedentary time and MCI, highlighting the importance of limiting sedentary behavior as older adults with MCI tend to have longer sedentary time, leading to worse health outcomes. Gafni et al. (74) found that insufficient PAL and sitting for at least three-quarters of the day increased MCI risk in older adults. A systematic review and meta-analysis suggested (75) that older adults meeting the 24-h movement guidelines (i.e., ≥60 min of moderate-to-vigorous physical activity, ≤2 h of screen time, and age-appropriate sleep) experience better cognitive health outcomes.

Sedentary behavior may impact brain health and increase MCI risk through multiple direct and indirect biological pathways (76–78). Prolonged sitting impairs vascular endothelial function, reduces cerebral blood flow, and diminishes cerebrovascular reserve. Studies show prolonged sitting significantly reduces middle cerebral artery blood flow velocity (79, 80). Chronic cerebral hypoperfusion, a common pathological basis for vascular cognitive impairment and AD, affects neuronal energy supply, accelerates Aβ deposition and tau hyperphosphorylation, thereby impairing cognition. Sedentary behavior is a key contributor to insulin resistance, type 2 diabetes, and dyslipidemia. These metabolic disturbances trigger systemic inflammation and oxidative stress, disrupt blood–brain barrier integrity, and directly damage neuronal and synaptic function. Brain insulin resistance is a core pathological mechanism in AD (termed “type 3 diabetes”), closely linked to impaired Aβ clearance and tau pathology (81). Sedentary behavior is associated with elevated circulating pro-inflammatory cytokines (e.g., IL-6, TNF-*α*, CRP) (82, 83). Chronic low-grade inflammation is a key driver of neurodegeneration. These inflammatory factors can cross the impaired blood–brain barrier, activate microglia, induce neuroinflammation, and ultimately lead to impaired synaptic plasticity and neuronal death (84).

Physical activity, particularly moderate-to-vigorous activity, effectively elevates BDNF levels, crucial for neuronal survival, differentiation, learning, and memory. Conversely, sedentary behavior may downregulate BDNF. An intervention study showed that even short-term (e.g., one week) strict bed rest led to a significant drop in serum BDNF and slightly worsened cognitive test performance in healthy adults (37). Furthermore, sedentary behavior may indirectly affect brain health through impacts on lipid metabolism (leading to obesity), disruption of circadian rhythms, and reduced social and cognitive stimulation. These intertwined mechanisms form a complex network through which sedentary behavior impairs cognitive function (85).

This study has several limitations: (1) The cross-sectional design cannot establish causality between PAL, sedentary time, and cognitive changes. (2) The sample was from Xinjiang, limiting generalizability. (3) PAL and sedentary time were self-reported, susceptible to recall bias. Future research should employ longitudinal designs and objective measures like accelerometers to further investigate specific parameters of physical activity (intensity, frequency, pattern) and optimal strategies for interrupting sedentary behavior to provide more precise public health guidance.

In summary, both physical activity and sedentary time are closely associated with MCI incidence in older adults. Maintaining a weekly PAL between 1,485 and 4,000 MET-min/wk. and limiting daily sedentary time to under 200 min may help reduce the risk of MCI.

## Data Availability

The original contributions presented in the study are included in the article/supplementary material, further inquiries can be directed to the corresponding author.
